# Assessment of genetic diversity, population structure, and gene flow of tigers (*Panthera tigris tigris*) across Nepal's Terai Arc Landscape

**DOI:** 10.1371/journal.pone.0193495

**Published:** 2018-03-21

**Authors:** Kanchan Thapa, Sulochana Manandhar, Manisha Bista, Jivan Shakya, Govind Sah, Maheshwar Dhakal, Netra Sharma, Bronwyn Llewellyn, Claudia Wultsch, Lisette P. Waits, Marcella J. Kelly, Jean-Marc Hero, Jane Hughes, Dibesh Karmacharya

**Affiliations:** 1 Department of Fish and Wildlife Conservation, Virginia Tech, Blacksburg, Virginia, United States of America; 2 Center for Molecular Dynamics Nepal, Thapathali-11, Kathmandu, Nepal; 3 Department of National Parks and Wildlife Conservation, Kathmandu, Nepal; 4 Environment Team, U.S. Agency for International Development, Kathmandu, Nepal; 5 American Natural History Museum, New York City, New York, United States of America; 6 Department of Fish and Wildlife Sciences, University of Idaho, Moscow, Idaho, United States of America; 7 School of Environment, Griffith University, Nathan, Queensland, Australia; Centre for Cellular and Molecular Biology, INDIA

## Abstract

With fewer than 200 tigers (*Panthera tigris tigris*) left in Nepal, that are generally confined to five protected areas across the Terai Arc Landscape, genetic studies are needed to provide crucial information on diversity and connectivity for devising an effective country-wide tiger conservation strategy. As part of the Nepal Tiger Genome Project, we studied landscape change, genetic variation, population structure, and gene flow of tigers across the Terai Arc Landscape by conducting Nepal’s first comprehensive and systematic scat-based, non-invasive genetic survey. Of the 770 scat samples collected opportunistically from five protected areas and six presumed corridors, 412 were tiger (57%). Out of ten microsatellite loci, we retain eight markers that were used in identifying 78 individual tigers. We used this dataset to examine population structure, genetic variation, contemporary gene flow, and potential population bottlenecks of tigers in Nepal. We detected three genetic clusters consistent with three demographic sub-populations and found moderate levels of genetic variation (H_e_ = 0.61, A_R_ = 3.51) and genetic differentiation (F_ST_ = 0.14) across the landscape. We detected 3–7 migrants, confirming the potential for dispersal-mediated gene flow across the landscape. We found evidence of a bottleneck signature likely caused by large-scale land-use change documented in the last two centuries in the Terai forest. Securing tiger habitat including functional forest corridors is essential to enhance gene flow across the landscape and ensure long-term tiger survival. This requires cooperation among multiple stakeholders and careful conservation planning to prevent detrimental effects of anthropogenic activities on tigers.

## Introduction

Reduction in prime habitat and loss of genetic diversity are influential factors leading to the extirpation of wildlife populations [1] and extinction of species [2–4]. In the face of habitat fragmentation and isolation, maintaining genetic connectivity across fragmented landscapes is challenging [5, 6] yet necessary to avert the negative consequences of genetic drift and inbreeding [7–10]. Maintenance of genetic diversity and gene flow is particularly critical for large carnivores, which often occur at naturally low densities [11], thus increasing their risk of extinction due to a greater susceptibility to stochastic events [12, 13].

The tiger (*Panthera tigris)* is a species of global conservation concern as its range has declined more than 90% in past two decades, and the species now occupies only 7% of its historic range [14]. The few remaining tigers (est. 3,200) are concentrated within 76 global Tiger Conservation Landscapes (TCLs) spread across a wide range of tiger habitat types [14]. Besides poaching, habitat loss and fragmentation remain the largest threats to the survival of extant tiger populations [15], and threats are mounting in these TCLs [14, 16]. One key region for global tiger conservation is the Terai Arc Landscape (TAL),which occupies a significant portion (~29,100 km^2^) of the Eastern Himalayan eco-region [17] and includes 15 core tiger clusters (identified as Protected Areas) connected by contiguous forest blocks in Nepal and northwest India [18]. Five protected areas with varying degrees of structural connectivity are located in Nepal, and are spread in a somewhat linear configuration across primarily forested habitat containing Nepal’s only tiger populations (N = 198) [19]. Maintaining functional connectivity for tigers in this region is crucial for preserving genetic variation and long-term population viability [20, 21]. Previous studies of genetic diversity and structure among tigers have shown that Bengal tigers (*Panthera tigris tigris*) are the most diverse globally and represent half of the extant genetic diversity in the species [22]. Multiple studies in India detected moderate to high levels of genetic diversity and varying levels of gene flow between the tiger sub-populations living in fragmented and human-altered landscapes [22–27]. Three possible demographic sub-populations of tigers have been identified across the Terai Arc Landscape of Nepal based on long-term field data and tiger habitat requirements [17, 28], and some degree of functional connectivity is expected across this region [29]. A recent study has shown that tigers occupy 36% of the TAL in Nepal and that core tiger populations occur within the protected areas [30]. Few signs of tigers have been detected outside of protected areas [29]. However, radio-telemetry studies have shown that tigers in the Terai have dispersed as far as 30 km [31], including through human dominated areas within the landscape [25, 26], and camera trap data have confirmed the presence of tigers in corridors [32]. The TAL has experienced significant landuse changes in the recent past [33] that might impede dispersal and gene flow across the landscape and create genetic subdivision. Thus, a landscape-level genetic study is needed to assess whether dispersal and subsequent breeding (genetic migration) of tigers occurs across this fragmented region.

**Fig 1:**
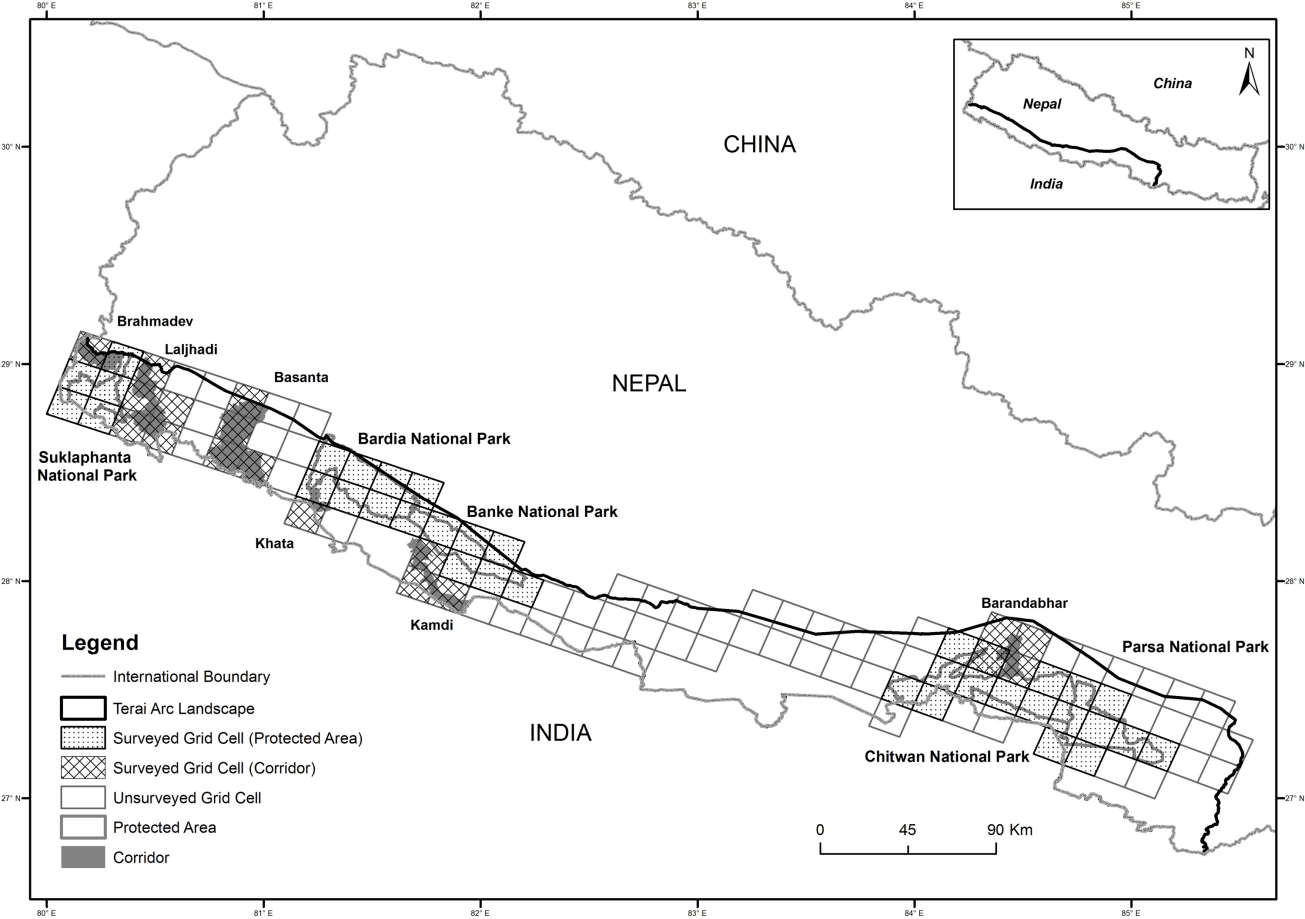
Study area and sampling grids cells (15 X 15 km) used for collection of tiger scat samples across the Terai Arc Landscape, Nepal. We searched and collected tiger scats (*n* = 770) from 54 grid cells (Protected Areas: 36 grids; Corridors: 18 grids) out of total 108 grid cells totaling 9,000 km^2^ of land area.

We conducted a forest change analysis in the Nepalese portion of the TAL (Fig 1) to identify major changes in land use (forest to agriculture) and implemented the first comprehensive fecal based non-invasive genetic assessment of tigers across the country. We focused our field sampling in 5 protected areas and six putative forest corridors, which represent the core area for tigers within the TAL-Nepal. Our main study objectives were to: 1) document the scale and distribution of land use change over the last 300 years in the TAL landscape, 2) determine the number of genetic groups of tigers, 3) assess genetic diversity, population structure and gene flow, 4) determine the level of contemporary migration between genetic groups, and 5) test for evidence of population bottlenecks. We hypothesized that tigers would group into three genetic clusters representing the three demographic tiger populations previously identified in TAL-Nepal [34]. Given the high degree of land use change in the past, we expected low to moderate levels of genetic diversity; and limited but detectable gene flow within the region.

## Materials and methods

### Ethics statement

All the tiger scat samples were collected non-invasively without capturing or handling any animal. The study permit was granted by the Department of National Parks and Wildlife Conservation, Ministry of Forest and Soil Conservation, Government of Nepal, Kathmandu, Nepal (Letter No. 403-08/09/2010).

### Study area

This study was conducted across the TAL-Nepal landscape (23,199 km^2^) that stretches along the southern lowland areas of the country (Fig 1). The TAL has a sub-tropical monsoonal climate and mixed deciduous vegetation ranging from successional alluvial floodplain grasslands communities to Climax Sal (*Shorea robusta*) forests. This global priority landscape [35] includes five protected areas: Suklaphanta National Park (SuNP), Bardia National Park (BNP), Banke National Park (BaNP), Chitwan National Park (CNP), and Parsa National Park (PNP) (Fig 1). Nepal’s protected areas are also connected across the border with TAL region of India's 10 protected areas [18]. Based on previous camera trap studies, adjacent CNP-PNP has the highest tiger abundance and SuNP the lowest [19].

### Forest-agriculture land-use changes

Land-use changes in the past 400 years across the TAL were analyzed using the Anthrome 2.0 datasets [36]. Anthrome data sets characterize global anthropogenic transformation at the century level (i.e., every 100 years) for the terrestrial biosphere in a discrete time frame for the years 1700–2000. We clipped the coarse data sets (~10 km pixels size) for the landscape using ArcGIS 10.1 (Esri, Redlands, USA) and calculated the amount of area under different land-use classes across different time frames (1700–1800–1900–2000). Thirteen land-use classes were classified from Ellis et al. [36] into two classes (forest and agriculture-settlement) to analyze the changes from forest to agriculture or vice versa over the time frame. This analysis was performed to identify major changes in land cover, which was used to develop hypotheses about potential movement barriers and bottleneck events impacting Nepal’s tiger population over time.

### Fecal DNA sampling

Putative tiger fecal (scat) samples were collected from protected areas and connecting wildlife corridors across the TAL-Nepal. We divided the study area into 108 grid cells each measuring 15 X 15 km (225 km^2^, sampling unit) and surveyed 54 grid cells (Fig 1) that had the high probability of tiger occurrence based on the Barber-Meyer et al [37] occupancy analysis. These selected grid cells covered five protected areas and six corridors. Within each grid cell, fecal samples were detected and collected during opportunistic field surveys. Field teams followed human trails, fire lines, and animal trails. Fecal samples were field-identified based on their physical appearance and associated indirect tiger signs like pugmarks and scratches [38].

A few grams from the upper surfaces of the scat were removed [39] and stored at room temperature in sterile 2-ml vials filled with DETs buffer (dimethyl sulphoxide saline solution) [40] at1:4 volume scat-to-solution ratio. The remaining fecal sample was left in the original location to minimize disturbance on any tiger signs. Scat samples were transported to the Kathmandu-based laboratory of the Center for Molecular Dynamic Nepal (CMDN) for genetic analysis.

### DNA extraction, species identification and sex identification

DNA was extracted from scat samples using a commercially available QIAmp DNA Stool Kit (QIAGEN Inc., Germany) following the manufacturer’s instructions. Species identification polymerase chain reaction (PCR) was performed using tiger-specific primers (TIF/TIR) (S1 Table) [41] that amplify a 162 bp mtDNA cytochrome b fragment. The genetically identified tiger samples were further processed for sex identification (S1 and S2 Tables) [42].

### Microsatellite primer selection and genotyping for individual identification

Sixteen microsatellite markers used by previous studies [41, 43–45] were evaluated. Based on amplification success and degree of polymorphism, we amplified 10 polymorphic microsatellite loci but retain only 8 microsatellite loci for individual identification and genetic analyses. (S3 Table).

PCR amplification was carried out in a 7 μL reaction volume containing 3.5 μL of Qiagen master-mix (Qiagen Inc., Germany). The PCR conditions for microsatellites amplification included an initial denaturation (95°C for 15 min) with a touchdown PCR step for 10 cycles (denaturation at 94°C for 30 s, annealing initially at 62°C and reduced by 0.5°C in each cycle for 90 s and extension at 72°C for 60 s). This was followed by 25 cycles of denaturation at 94°C for 30 s, annealing at 57°C for 90 s and extension at 72°C for 60 s, and a final extension at 72°C for 10 mi. PCR product (0.7 μL) were sized against LIZ-500 size standard in ABI 310 genetic analyzer (Applied Biosystems, USA). Three to five PCR replicates were run per sample. Microsatellite alleles were scored in GENEMAPPER, version 4.1 (Applied Biosystems, USA). To finalize consensus genotypes, at least three identical homozygote PCR results were assessed to determine homozygote, and each allele was observed in two independent PCR for a heterozygous genotype [46].

### Genetic analyses

PCR amplification success rates and genotype accuracy (GA) were based on results from the last two PCR runs [46]. PCR amplification success was based on the percentage of PCR successes across all the tiger-positive samples. Genotype accuracy was calculated based on the percentage of successful PCR results that matched the finalized consensus genotype. In addition, cumulative probabilities of identity for unrelated individuals (P_(ID)_) and siblings (P_(ID)sib_) were estimated using Gimlet, version 1.3.3[47]. A minimum criteria of P_(ID)sib_ [48, 49] for selecting the minimum number of loci required for individual identification was set at 0.035. The GenAlEx program, version 6.5 [50] was used to assess genotype matching and determine the minimum number of individual tigers in the consensus genotype data sets.

Genetic diversity was quantified by estimating observed heterozygosity (H_o_) and expected heterozygosity (H_e_) using GenAlEx, version 6.41[50]; and allelic richness using the rarefaction method in HP-RARE, version 1.0 [51]. Global and population-level deviations from the Hardy-Weinberg equilibrium (HWE) and linkage disequilibrium (LDE) [52] were calculated using ARLEQUIN, version 3.5 [53] and evaluated with and without Bonferroni corrections for multiple tests [54]. ARLEQUIN was also used to estimate F_ST_, inbreeding coefficients (F_IS_) and to test the statistical significance of the pair-wise F_ST_ values between the populations in the TAL, using 10,000 permutations [55]. This was further complemented by the Analysis of the Molecular Variance (AMOVA), implemented within ARLEQUIN, was used to assess the amount of variation within, and across, individuals and populations (CNP, BNP, SWR) in TAL. D_EST_ was used as an alternative metric for genetic distances between the populations, as it outperforms F_ST_ as an accurate and unbiased metric of the level of differentiation between populations when the sample size is high and the number of loci is low [56]. The harmonic mean of D_EST_ across the loci for each population was calculated using the web-based program SMOGD [57] with 1,000 bootstrap replicates.

To visualize genetic similarities among regions and individuals, we performed a multivariate principal coordinate analysis (PCoA) in GenAlEx. Furthermore, an individual-based Bayesian clustering approach was implemented using STRUCTURE (non-spatially explicit), version 2.3.4 [58] and TESS (spatially explicit), version 2.3[59], for inferring genetic subdivision across the tiger population in the TAL. In STRUCTURE, the value *k*, representing the potential number of genetic clusters (sub-populations), was allowed to vary between 1 and 5; we performed 10 independent runs for each value of *k*. This analysis was run with admixture models with correlated frequencies using a burn-in of 500,000 Markov chain Monte Carlo (MCMC) steps followed by an additional 1,000,000 iterations without prior information on the sampling sites. The optimal value of *k* was inferred by examining the likelihood curve, q value bar plots, and using the Evanno method [60] implemented in the web-based program Structure Harvester [61]. The individual membership assignments estimated in STRUCTURE were analyzed in program CLUMPP [62] with a greedy algorithm and 10,000 random permutations for estimating the mean of the permuted matrices across replicates. We used the stringent criterion of q> 0.8 for assigning individuals as residents to potential sub populations. Values below 0.8 were considered representative of individuals with admixed ancestry.

The admixture models (convolution Gaussian model [BYM] and the conditional auto-regressive [CAR]) were run using TESS [63] with 100,000 burn-in runs followed by 20,000 iterations for *k* = 2 to 10 with 100 replications per *k*. The average of the 10% of the lowest Deviance Information Criteria (DIC) values was used for each *k*_max_. DIC values were taken for estimating the number of optimal *k*_max_ (genetic sub-populations) [64]. DIC values averaged over100 independent iterations were plotted against *k*_max_ and most likely value of *k*_max_ was selected by visually assessing the point at which DIC first reached a plateau and the number of clusters to which individuals were proportionally assigned.

We also examined isolation-by-distance (IBD) and spatial auto-correlation patterns to characterize spatial genetic structure of tigers in TAL using GenAlEx. First, we tested whether a significant correlation existed between pair-wise co-dominant genotypic distance and geographical distance by applying simple Mantel tests with 9,999 permutations[65]. Secondly, we used spatial auto-correlation analysis to test the spatial extent of the genetic structure against the null hypothesis of no auto-correlation (correlation coefficient, *r* = 0) by generating 95% confidence intervals (CI) for each distance class via permutation (9,999 simulations) and bootstrapping (999 repeats). We correlated genetic distances and spatial distance matrices and generated auto-correlation matrices for each spatial distance class ranging from 0–250 km based on the distribution of tigers in TAL. Results were visualized as correlograms and the location of first x-intercept represents the extent of non-random spatial genetic structure. Individuals below this threshold share a higher proportion of genes than spatially distant individuals. Within a given correlogram, significant spatial auto-correlation was confirmed only when a positive *r*-value fell outside the 95% CI (derived from the permutation test), and when the 95% CI about *r* (derived from bootstrapping) did not intercept the axis of *r* = 0[66].

To examine whether the individuals were born in the location from which they were sampled, assignment/exclusion tests were performed in GENECLASS, version 2.0 [67]. The Bayesian approach with re-sampling algorithm [68] was used with 10,000 individuals at an assignment threshold (alpha, α) value of 0.01. The likelihood ratio test statistic (L_*home*_/ L_max_) was applied to identify migrants. This method uses the Bayesian criteria of Rannala & Mountain (1997) [69] along with the MCMC re-sampling method to determine the critical value of L_*home*_/ L_max_ beyond which a sample is treated as a migrant. We also carried out assignment tests in program STRUCTURE incorporating the geographical sampling sites as prior information (LOCPRIOR) without changing the other parameter settings as described above. Since we did not have prior knowledge about migration of individuals (MIGPRIOR) between potential sub-populations, we used the default setting.

Rates of recent immigration over the last several generations among the sub-populations were estimated using program BayesAss+, version 3.0 [70]. This Bayesian approach uses the multi-locus genotype data and relaxes the key assumption that populations are in HWE or migration-drift equilibrium. Recent gene flow (over the past 5–7 generations, approximately 25–30 years) was assumed [27], given a 7.55 year generation time for tigers [71]. Multiple runs (n = 5) of the program BayesAss+ with 3×10^7^ MCMC iterations and a 10^7^ burn-in with different seed numbers and delta values confirmed the final parameters and ensured their convergence. Both immigration and emigration rates between populations were considered as contemporary migrations.

We used two approaches to test for the genetic signature of a severe demographic contraction (i.e. bottleneck) in the tiger population across the TAL. First, we used test M-ratio [72] implemented in ARELQUIN. The M-ratio compares the number of alleles (k) with the allelic size range (r). Presence of a bottleneck signature in a population occurs when rare alleles are lost along with reductions in k faster than r. Low M-ratio values less than the threshold of 0.68 are thought to represent the presence of a bottleneck signature in the population [72]. Second, we used the Cornuet and Luikart [73] approach in program BOTTLENECK [74] for comparing the bottleneck signature in each population. This method tests for the departure from mutation-drift equilibrium based on heterozygote excess or deficiency (H_eq_). Simulations were performed under three mutation models: infinite allele model (IAM), single stepwise model (SSM), and two-phase model (TPM). The simulation values were then compared to real data values to obtain the distribution of H_eq_. For the TPM, we used the generic values of 0.95 and 0.12 for frequency of single-step mutations and variance, respectively [74]. A Wilcoxon sign-rank test was used to detect heterozygosity excess or deficiencies across loci. We also used a qualitative approach with the mode shift test to detect a population bottleneck. Recently bottlenecked populations show a mode shift in the distribution of allele frequencies such that alleles with very low frequency (less than 0.1) are less abundant than alleles that occur frequently.

## Results

### Landuse changes

**Fig 2:**
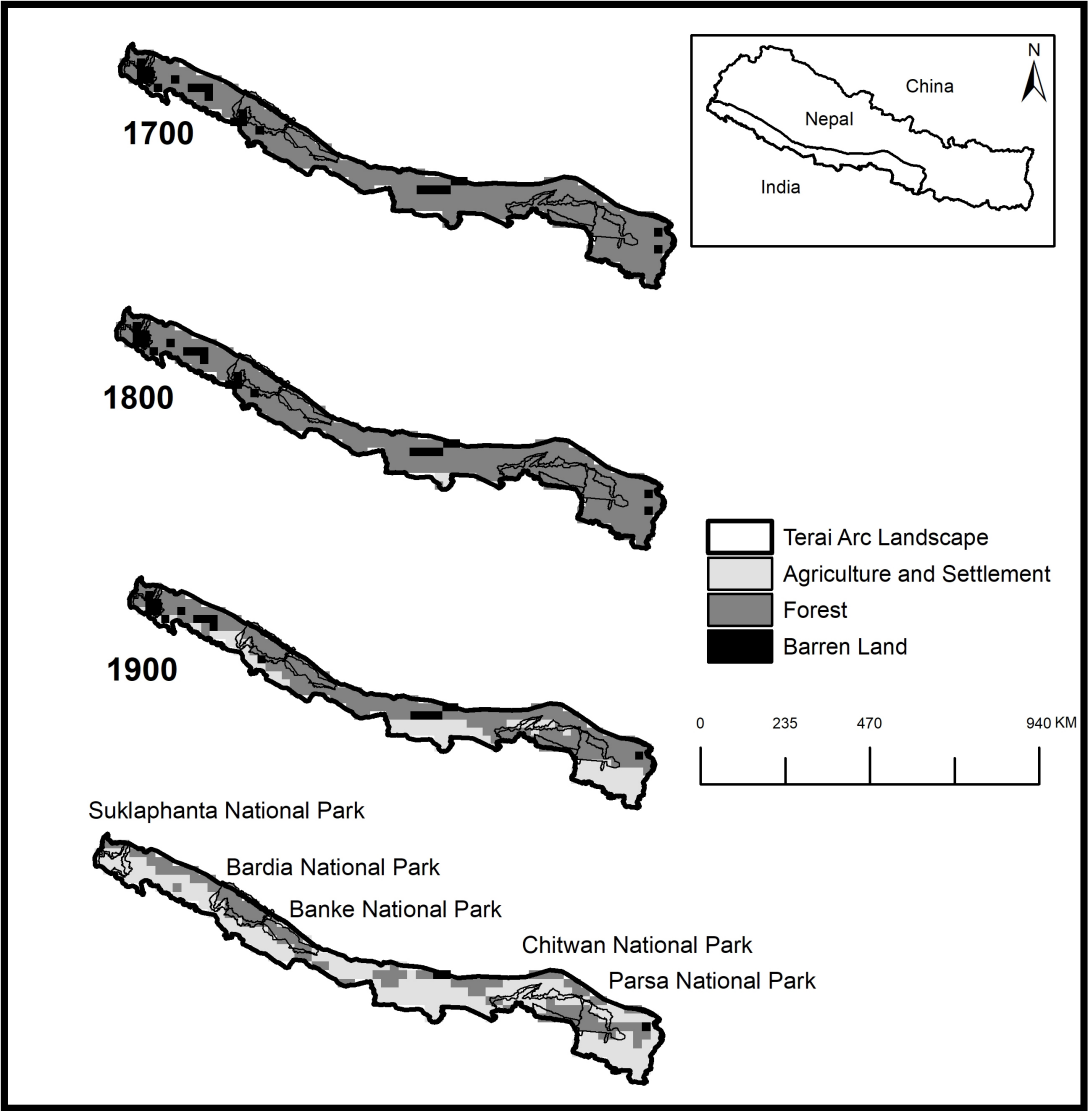
Land use change (forest into agriculture and settlement) analysis, in the last 400 years in the Terai Arc Landscape using Anthrome 2.0 datasets at resolution of ~10 km pixel size [36] in ArcGIS 10.1.

Land-use change analysis showed a dramatic decline in forest cover and an increase in agricultural areas by 62% in the last 300 years across the TAL-Nepal (Fig 2). The major decline in forested areas, with a 47% decrease in land cover, occurred between the 19^th^ and 20^th^ centuries. There has been a 97% increase in agriculture and settlement areas over the past 200 years in the TAL (S1 Fig). Within the TAL, a major break in the contiguous forest landscape occurred around 52 km west of Chitwan National Park due to a large-scale resettlement and development project between 1900-1960s. Among these protected areas, Chitwan valley (CNP and surrounding areas) suffered the largest and most dramatic decline in forested area. Therefore, we predicted that the CNP-PNP tiger population would be an isolated sub-population. Based on our landscape change analysis, we expected to find a bottleneck signature within CNP-PNP and the other hypothesized sub-populations (BNP and SuNP).

### Field sampling

In the first phase of the study (2010–2012), fecal samples were collected from grid cells (n = 36) within four protected areas (SuNP, BNP, CNP, and PNP). No fresh scats were found in Banke National Park. In the second phase (2012–2013), putative tiger samples were only found and collected in grids (n = 5) of the two known corridors (Khata and Basanta). No fresh tiger scats were found in corridors: Kamdi, Laljhadi, Barandabhar, and Bramahadev.

### Genetic analysis of tiger samples

**Fig 3:**
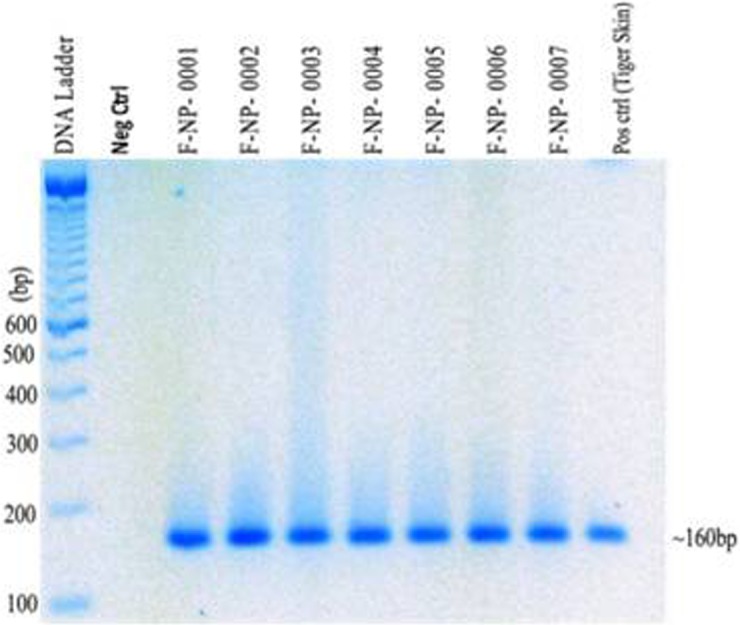
Tiger species identification using mtDNA PCR assay. Tiger positive samples yielded 162bp PCR fragments.

**Fig 4:**
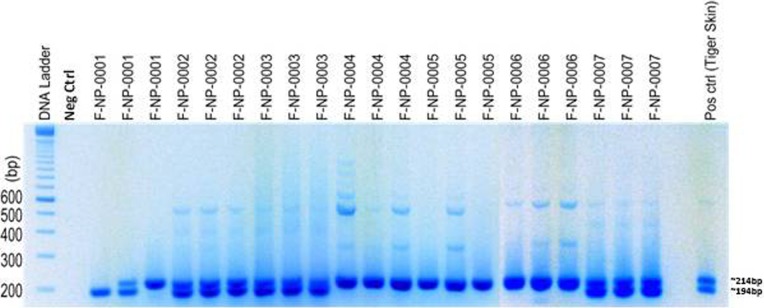
Sex identification of tiger species positive samples using Amelogenin gene PCR assay. Females have single band at 214 bp and males have bands at 194 bp and 214 bp.

Of 770 putative tiger scat samples collected from four protected areas (SuNP, BNP, CNP, and PNP) and two corridors (Khata and Basanta) within the TAL, a total of 412 were confirmed to be tiger scat (Fig 3) (Table 1). Of these, sex was genetically identified in 353 scat samples, among which 255 samples came from males and rest (98) were from females (Fig 4) (Table 1). Of the ten microsatellite loci that were amplified, eight were retained for individual identification and genetic analysis based on high PCR amplification success (84%), genotyping accuracy (82%) (Table 2), and polymorphism. Two Loci (FCA205 and PttA2) were removed from the analysis due to poor amplification success or monomorphism. For the remaining 8 loci, the cumulative P_(ID)_ and P_(ID)sib_ were estimated to be 1.5E-06 and 3.2E-03 (<0.01) respectively, and 3.1 E-02 for our 6 least powerful loci. We obtained a consensus genotype at 6–8 loci for 212 samples (51% genotyping success) and identified 78 individual tigers (male = 49, female = 27, unknown sex = 2). Only four scat samples collected outside of protected areas were of tiger (Table 1). Unfortunately, we were unable to identify the number of individuals from these samples due to poor DNA quality. Only one tiger sample from the PNP collected 15 km east of the CNP was successfully genotyped and added to the CNP dataset.

**Table 1:** Summary of scat DNA analysis success rates for species and sex identification, and overall microsatellite genotyping based on putative tiger scat samples (n = 770) collected in the Terai Arc Landscape, Nepal. Species identification was based on the total number of samples processed; sex identification was based on the total number of tiger positive samples; and genotyping success was based on the number of positive samples for species and sex identification. M, male; F, female.

Study Areas	Total Samples Processed	Tiger Species Identification % (# samples)	Sex Identification % (# samples; M, F)	Genotyping Success % (# samples)
Chitwan National Park	420	61 (257)	93 (239; 185, 54)	56(145)
Bardia National Park	116	67 (78)	85 (66; 46, 20)	55(43)
Suklaphanta National Park	79	83 (66)	59 (39; 19, 20)	35(23)
Parsa National Park	85	8 (7)	85 (6; 4, 2)	14(1)
Corridors	70	6 (4)	75 (3; 1, 2)	0(0)
All Areas	770	54 (412)	86 (353; 255, 98)	51(212)

**Table 2:** Summary of PCR amplification success and genotyping accuracy (GA) for 8 microsatellite loci for all processed tiger samples (*n* = 401) detected across the three protected areas: Chitwan National Park, Bardia National Park, and Suklaphanta National Park across the Terai Arc Landscape, Nepal.

Locus	All Samples (*n* = 401)		Chitwan NP (*n* = 257)		Bardia NP (*n* = 78)		Suklaphanta NP (*n* = 66)	
	PCR	GA	PCR	GA	PCR	GA	PCR	GA
FCA391	75.7	88.6	87.6	89.5	57.5	89.9	81.9	86.4
PttD5	88.4	88.8	91.7	92.0	83.3	83.8	90.3	90.6
FCA232	87.5	77.0	82.8	80.2	82.5	65.6	97.2	85.3
FCA304	94.0	87.5	93.9	90.2	90.8	79.4	97.2	92.7
FCA043	93.6	80.3	95.2	89.0	92.5	80.6	93.1	71.2
F53	65.8	84.6	69.7	81.9	33.3	79.4	94.4	92.5
F85	73.9	73.4	75.5	85.1	89.2	57.1	56.9	78.0
FCA441	94.8	74.7	94.3	85.8	91.7	71.3	98.6	67.2
Mean	84.2	81.9	86.3	86.7	77.6	75.9	88.7	83.0
SD	11.0	6.3	9.5	4.2	21.2	10.6	13.9	9.8

PCR, % polymerase chain reaction amplification success; GA, % genotyping accuracy; NP: National Park; SD: Standard deviation

### Equilibrium analyses and genetic diversity

Based on the population-level analysis, four loci in CNP, two in BNP, and three in SuNP deviated significantly from Hardy-Weinberg Equilibrium (HWE) at *P* < 0.05 (Table 3). No locus was consistently out of the HWE across all sampling sites. After Bonferroni corrections, one locus (F85) in CNP and one locus (F53) in SuNP remained significantly out of the HWE. Significant linkage disequilibrium after sequential Bonferroni corrections (*P*≤ 5.95E-04) was detected among three pairs of loci with no apparent pattern (FCA232-FCA043 in CNP, FCA304-F53 and FCA391-FCA232 in SuNP). Overall average genetic diversity of the TAL tiger population was as follows: observed heterozygosity of 0.54, expected heterozygosity of 0.61, mean number of alleles per locus of 6.0, and mean allelic richness of 3.51. Levels of genetic variability varied among the protected areas, with the highest genetic diversity found in CNP and the lowest in SuNP (Table 3). The average local inbreeding coefficient was high (F_IS_ = 0.16) in the smaller SuNP population relative to other sites in the TAL (Table 3), which is consistent with the lower diversity observed for SuNP population.

**Table 3:** Genetic diversity estimates across 8 microsatellite loci for tigers studied in three protected areas across the Terai Arc Landscape, Nepal: Chitwan National Park, Bardia National Park, and Suklaphanta National Park.

Locus	Chitwan National Park (*n* = 37)						Bardia National Park (*n* = 25)						Suklaphanta National Park (*n* = 16)					
	N_A_	A_R_	H_o_	H_E_	*P*_HW_	F_IS_	N_A_	A_R_	H_o_	H_E_	*P*_HW_	F_IS_	N_A_	A_R_	H_o_	H_E_	*P*_HW_	F_IS_
FCA391	4.00	3.96	0.78	0.69	0.67	−0.11	3.00	2.95	0.26	0.42	0.00	0.39	3.00	2.78	0.31	0.51	0.10	0.41
PttD5	4.00	2.66	0.32	0.30	0.01	−0.07	4.00	3.75	0.54	0.53	0.93	−0.01	3.00	2.99	0.63	0.54	0.05	−0.12
FCA232	3.00	2.89	0.74	0.55	0.04	−0.35	5.00	3.84	0.39	0.40	0.79	0.03	3.00	2.56	0.13	0.12	1.00	−0.02
FCA304	5.00	3.62	0.65	0.51	0.11	−0.27	5.00	3.75	0.52	0.44	0.61	−0.16	2.00	2.00	0.19	0.48	0.03	0.63
FCA043	4.00	3.87	0.51	0.61	0.01	0.17	5.00	4.28	0.57	0.53	0.56	−0.04	4.00	3.77	0.38	0.54	0.07	0.33
F53	5.00	4.26	0.67	0.67	0.42	0.02	5.00	4.91	0.56	0.75	0.03	0.29	5.00	5.00	0.67	0.73	0.00*	0.13
F85	6.00	4.83	0.42	0.64	0.00*	0.36	4.00	3.50	0.63	0.61	0.90	0.00	3.00	3.00	0.73	0.64	0.91	−0.12
FCA441	4.00	3.30	0.57	0.58	0.89	0.03	4.00	3.99	0.92	0.72	0.54	−0.26	3.00	3.00	0.64	0.62	0.89	0.00
**Average**	**4.00**	**3.67**	**0.58**	**0.57**		**-0.03**	**4.00**	**3.87**	**0.57**	**0.55**		**0.03**	**3.00**	**3.14**	**0.46**	**0.52**		**0.16**
**SE**	**0.32**	**0.72**	**0.06**	**0.04**		**0.08**	**0.26**	**0.57**	**0.04**	**0.05**		**0.07**	**0.31**	**0.90**	**0.08**	**0.06**		**0.09**

*n*, sample size; N_*A*_, number of alleles; A_*R*_, allelic richness using the rarefaction method; H_*o*_, observed heterozygosity; H_*e*_, expected heterozygosity; P_*HW*_, *P* values for exact tests of Hardy-Weinberg equilibrium (level of significance, α = 0.05); F_*IS*_, inbreeding coefficient.

* represents locus out of HWE after Bonferonni correction at *P* = 0.002; SE, standard error.

### Genetic structure

The mean global F_ST_ value (level of differentiation) for the TAL was found to be moderate (F_ST_ = 0.14 ± 0.07). Results of AMOVA showed that genetic variation among the sites was 13.7%, while the residual variation among individuals within the sites was 86.3% (S4 Table) indicating a low level of differentiation among the populations for the target species. The pair-wise F_ST_ and D_EST_ values (between protected areas) were found to be low to moderate and statistically significant (Table 4, *P* < 0.05). Comparatively low F_ST_ and D_EST_ were found between the BNP and SuNP populations. In contrast, levels of genetic differentiation were high (F_ST_ = 0.21) for SuNP and CNP populations (Table 4).

**Table 4:** Pair-wise measures of the level of differentiation of tiger sub-populations in the Terai Arc Landscape, Nepal based on F_ST_[55] and D_EST_ (in parentheses) [56] (below the diagonal). Pair wise geographical distance (in km) between core population (above diagonal).

Population	CNP	BNP	SuNP
CNP	―	314	450
BNP	0.08 (0.07)	―	136
SuNP	0.21 (0.21)	0.14 (0.12)	―

All pair-wise F_ST_ values were significant (*P*<0.05) based on 10,000 permutations.

**Fig 5:**
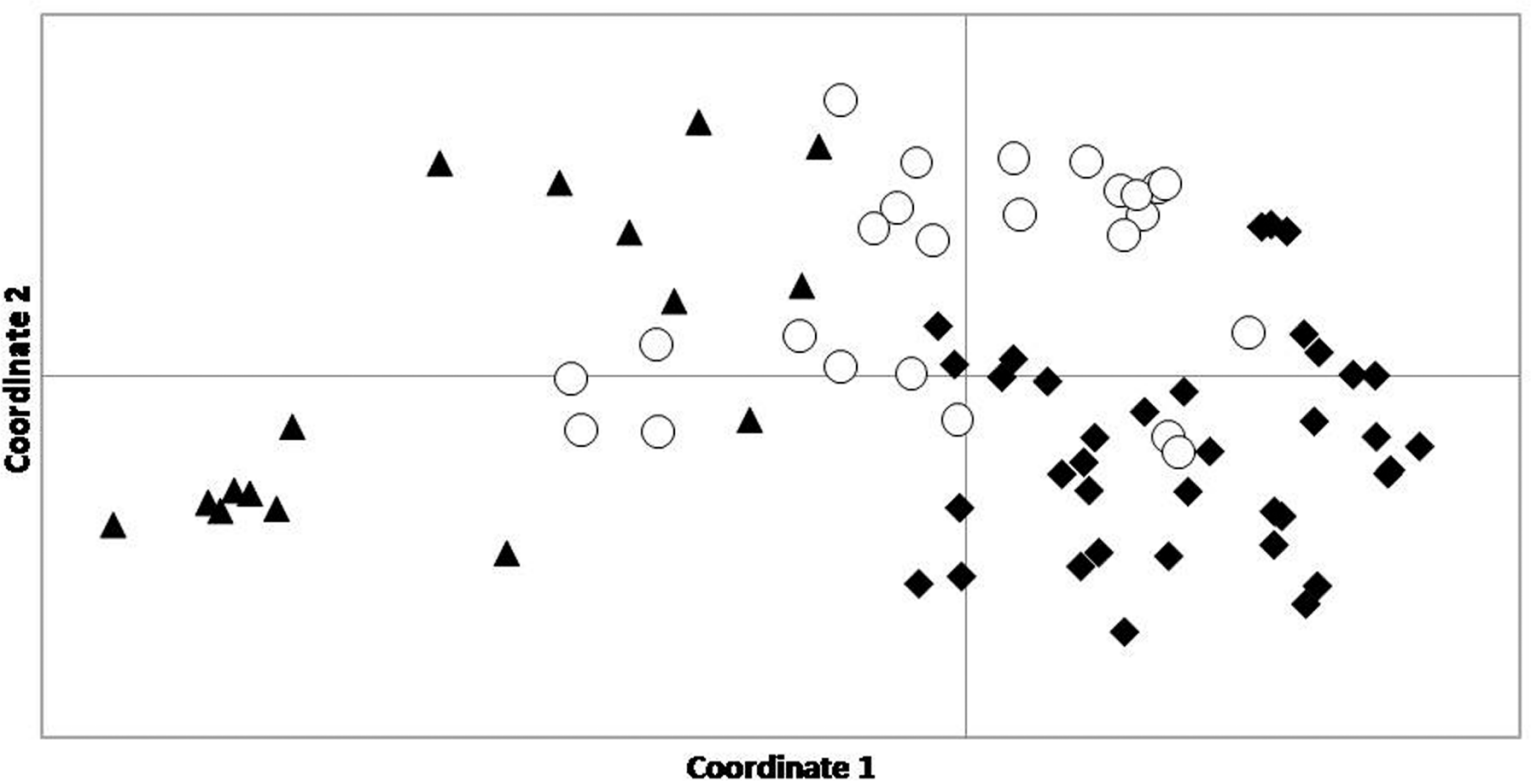
Principal Coordinate Analysis of tiger genotypes obtained from “♦” Chitwan National Park, “○” Bardia National Park, and “▲” Suklaphanta National Park, in the Terai Arc Landscape, Ne pal, assessed through program GenAlEx.

**Fig 6:**
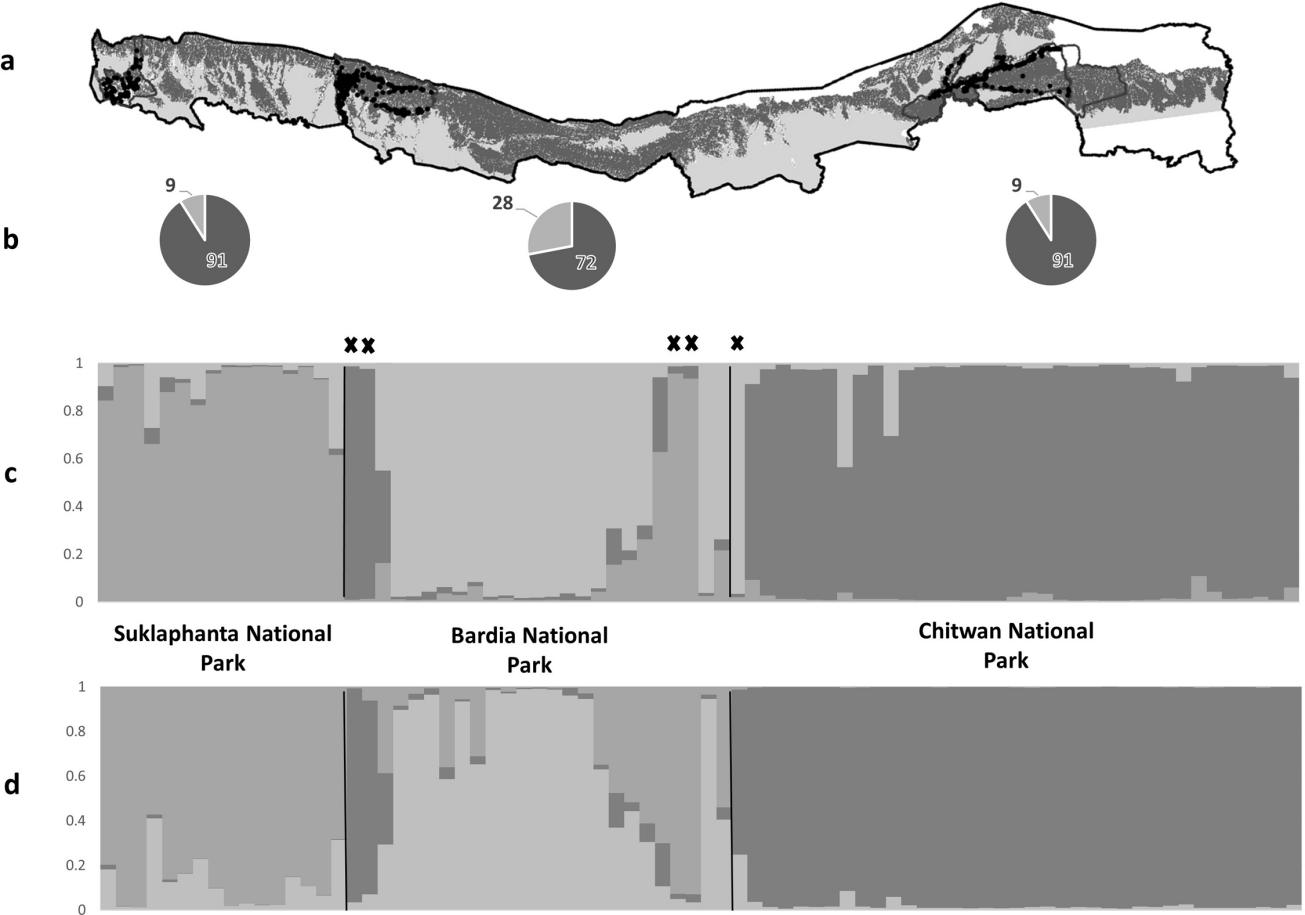
Map of the Terai Arc Landscape, Nepal. (a) Protected areas (starting from left: Suklaphanta National Park, SuNP; Bardia National Park, BNP; Chitwan National Park, CNP; along with the spatial location of identified tiger-positive samples (black dots). (b) Pie charts showing the percentage of ancestry assigned to other identified genetic clusters in the populations (orange), and the resident population (blue). (c) STRUCTURE (non-spatially explicit) bar plot with each bar representing an individual tiger (*n* = 78) in three populations across the Terai Arc Landscape revealing three (*k* = 3) admixed sub-populations (represented by 3 different colors) along with five migrants (marked as “*’) identified across the population. (d) Bar plot showing three identified sub-populations analyzed in spatially-explicit assignment program TESS.

The principal coordinate analysis performed in GenAlEx 6.5 showed that individual tigers formed three not so distinct overlapping clusters matching their sampling localities (CNP, BNP, SuNP) (Fig 5). Results of the Bayesian clustering analysis in STRUCTURE indicated three discrete, core populations across the TAL with some genetic admixture. The models showed highest statistical support at *k* = 4 based on the Ln P(*k*), and *k* = 3 based on delta *k* method. Four clusters showed a high standard deviation relative to *k* = 3 (S2 Fig) and sub-structure within CNP (S3 Fig). Hence, *k* = 3 was interpreted as the most likely value for the analysis, and it aligned with the prior knowledge of the spatial distribution of tiger demographic populations and land-use analysis (Fig 6). At *k* = 2, a major split was detected between CNP and the other two protected areas (BNP and SuNP, S3 Fig), which is consistent with the land use data and spatial separation of these sites (Figs 1 and 2).

**Fig 7:**
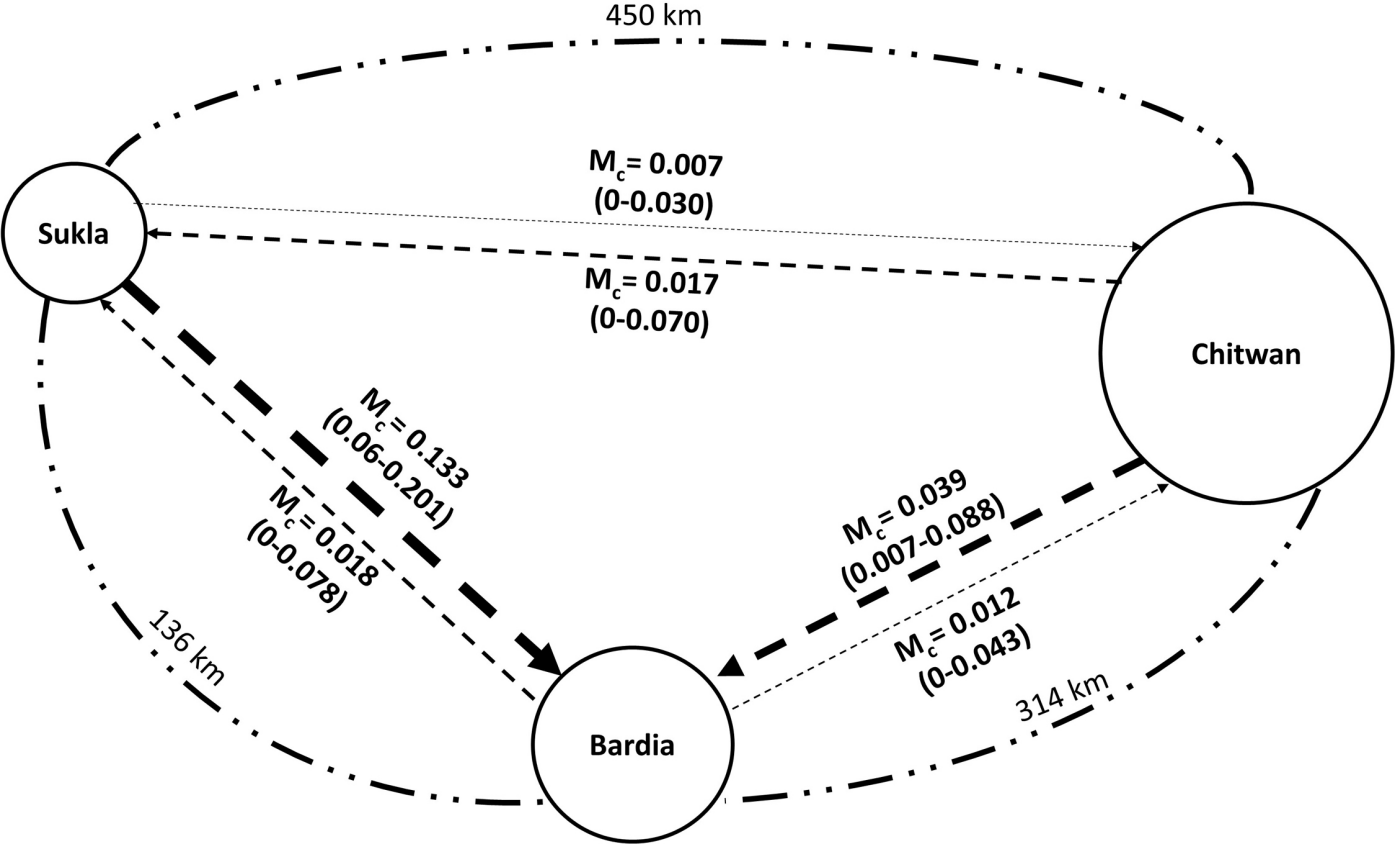
Contemporary gene flow patterns for tigers inferred across the Terai Arc Landscape, Nepal, based on migration rates (M_c_) estimated in BayesAss+[70]. Dashed lines in the center indicate direction of migration and line thickness represents the magnitude of estimates along with the migration rates. Figures within parentheses represent 95% CI for migration rates. Size of the circle represents the estimated size of breeding population. Broken lines around the periphery represent the spatial distances between the populations.

The inferred degree of admixture using STRUCTURE within populations was found to be variable across the three clusters (CNP, BNP, and SuNP) with the central cluster (BNP) showing the highest degree of admixture: 1^st^ cluster (91% CNP, 7% BNP, and 2% SuNP); 2^nd^cluster (13% CNP, 72% BNP, and 15% SuNP); and third cluster (2% CNP, 7% BNP, and 91% SuNP) (Fig 6). Within inferred clusters, sites (protected areas) are spatially separated from each other (1^st^- 2^nd^ clusters: 314 km; 2^nd^ and 3^rd^clusters: 136 km; and 1^st^ and 3^rd^ cluster: 450 km), with variable forest connectivity between them (S5 Table, Fig 7).

Results from the non-spatially explicit clustering analysis in STRUCTURE were corroborated with the spatially explicit analyses in TESS. Based on the DIC model selection criteria, graphs tended to plateau at *k* = 3, and the standard deviation increased with an increasing value of *k*_max_ (S4 Fig). The hard-clustering algorithm for individual membership with BYM admixture models did not change with *k*_max_≥ 3, while in the CAR admixture model, there were inconsistent results with *k*_max_> 3. Overall, *k* = 3 was the best supported model, inferred number of genetic clusters for the tiger populations across the TAL, thereby identifying CNP (1), BNP (2), and SuNP (3) as three distinct sub-populations with some evidence for sub-structure within CNP.

**Fig 8:**
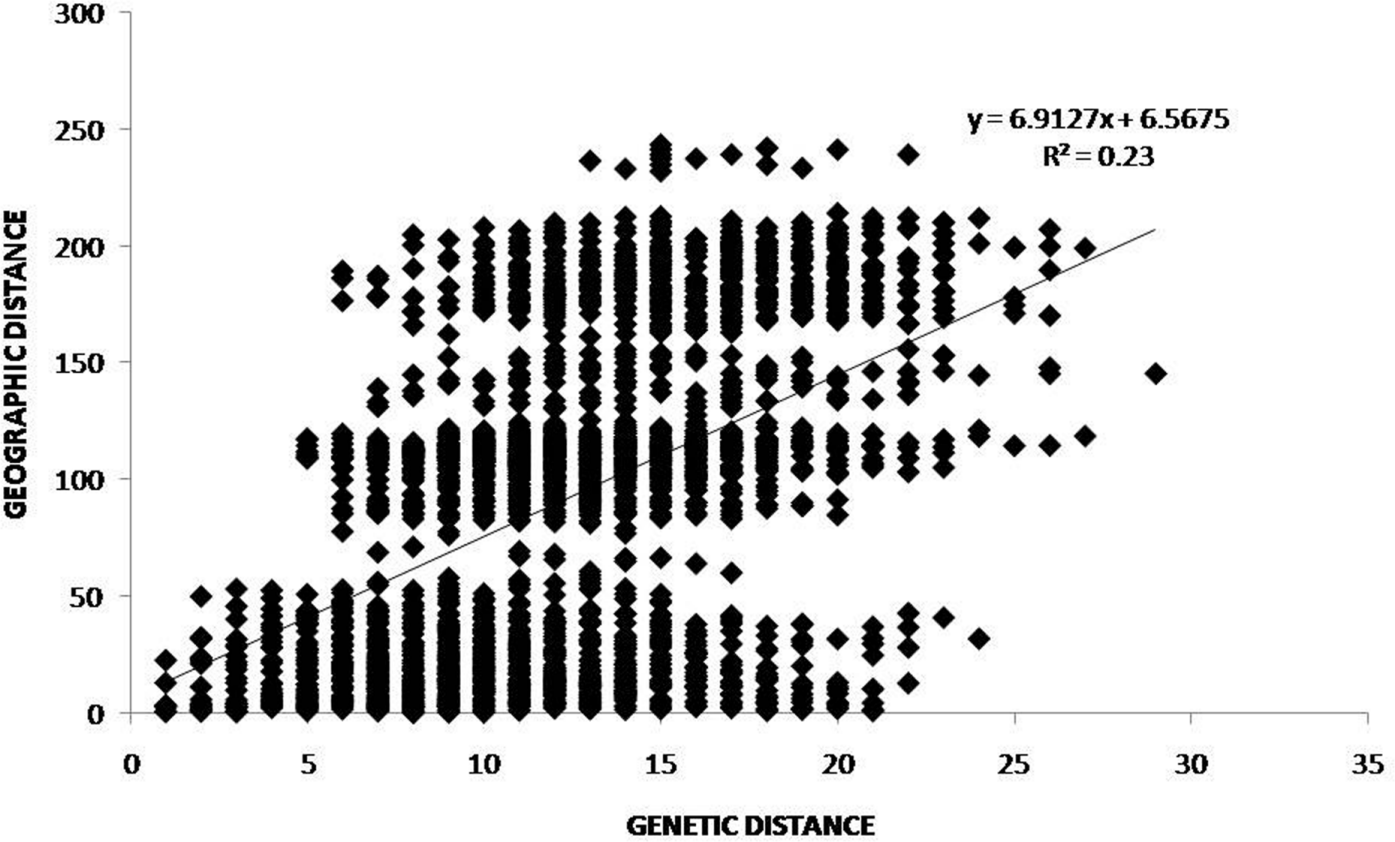
Isolation-by-distance patterns for tigers in Terai Arc Landscape, Nepal assessed by plotting pairwise codominant genotypic distance versus pairwise geographic distances (km) including statistical significance using simple Mantel tests in GenAlEx, version 6.5. Each point (diamond) represents a pair-wise comparison among individual tigers.

There was significant correlation (*r* = 0.48, *P* = 0.0001) between geographical distance and genetic distance (Fig 8), supporting the hypothesis of isolation-by-distance for tigers in the TAL. The auto-correlogram for all tigers showed significantly positive autocorrelation within 75 km distance class (25 km, *r* = 0.050, *P* = 0.0001; 50 km, *r* = 0.094, *P* = 0.0001; 75 km, *r* = 0.076, *P* = 0.02). An x-intercept of *r* at ~ between 75–100 km (S5 Fig), empirically shows presence of nonrandom spatial structuring and genetic association among individuals at distances <94 km.

### Detection of migrants

We identified a total of seven migrants across the TAL using our criteria. STRUCTURE identified five migrants, while Geneclass2 identified four migrants and BayesAss+ identified six (Table 5). Three migrants were identified by all three methods–one from BNP to CNP (male), and two from CNP to BNP (male). Two female tigers were classified as migrants by both STRUCTURE and BayesAss+ moving from SuNP to BNP. Geneclass2 and BayesAss+ identified two additional migration events (both male tigers) moving from the BNP to CNP and the SuNP to BNP. STRUCTURE results suggested that both male tigers had admixed genetic ancestry (Table 5). No migrants were detected between the CNP and SuNP sub-populations.

**Table 5:** First-generation migrants between the 3 main core tiger populations in the Terai Arc Landscape-Nepal detected using programs STRUCTURE, Geneclass2 and BayesAss+; *P* value from Geneclass2.

Tiger ID*	Sampling Location	*P* Value	STRUCTURE (*estimated ancestry from sampling locations)*	Assigned Population	Sex
CHIT001^(S,G,B)^	CNP	0.0001	0.03	BNP	Male
CHIT011^(G)^	CNP	0.009	0.94	BNP	Male
BRD001^(S,G,B)^	BNP	0.002	0.14	CNP	Male
BRD002^(S, G,B)^	BNP	0.007	0.21	CNP	Male
BRD023^(B)^	BNP	0.04	0.62	SuNP	Male
BRD024^(S, B)^	BNP	0.53	0.23	SuNP	Female
BRD025^(S,B)^	BNP	0.53	0.24	SuNP	Female

*Superscript caption with ID number indicates that individuals were identified as migrants either by STRUCTURE (S) or Geneclass2 (G) or BayesAss+ (B).

The BayesAss+ analysis showed symmetric migration between pairs of populations (Fig 7), except for SuNP. A high immigration rate was determined from the SuNP into the BNP sub-population (m_c_ = 0.13). The BNP sub-population appeared to be receiving the migrants from both CNP and SuNP, with a total migration rate of 0.17 (Fig 7). The 95% confidence intervals overlapped for migration rates for the pair of populations (SuNP-CNP; BNP-CNP) in both directions, suggesting roughly symmetric bi-directional gene flow between sub-populations. This did not hold for gene flow between BNP and SuNP, which appeared to be uni-directional from SuNP to BNP. The net emigration rate was highest for SuNP, as it contributed the most migrants to the other protected areas (Fig 7). The net emigration rate for BNP and CNP were negative, suggesting the site received more migrants than it contributed to other populations.

### Detection of bottlenecks

The average M-ratio describing potential bottlenecks for all sites was 0.29 (SE = 0.07), which is below the threshold value of 0.68. This suggests that the tiger population suffered a bottleneck event that caused a severe decline in the population size in the recent past. These results were supported among all sub-populations. The Bottleneck software detected heterozygote excess using the sign test, with 5 to 6 loci, depending upon the mutation models. However, results of the two-tailed Wilcoxon signed-rank test showed that the signature of a bottleneck event was significant only for the CNP population under the Infinite Allele Mutational (IAM) model (*P* = 0.03, S6 Table). The mode-shift test showed the normal “L”-shaped allele distribution in BNP. The test only showed the presence of a large proportion of alleles at low frequencies, indicating a genetic bottleneck between CNP and SuNP, but not CNP and BNP.

## Discussion

### Effect of landuse change

The historical land-use change analysis was consistent with the present structuring of animals on the landscape into sub-populations [27, 75, 76]. Tigers used to roam the vast expense of Terai forests in Nepal and India. Beginning in the mid-1840s, core tiger habitat was protected by the Rana rulers for their exclusive royal hunting, thus discouraging people from settling and conducting agriculture. Tigers were persecuted in large numbers during organized royal hunts. However, the population recovery of tigers was relatively quick following this time, likely due to immigration from adjacent forest areas. Rampant malaria also hindered people from clearing forest and settling in the Terai region [77].

By the late 20^th^ century however, extensive land clearing had occurred and the level of wildlife hunting was high, fragmenting tiger habitat, reducing abundance of tiger prey species, and contracting tiger range to the Chitwan Valley in central Nepal [75, 78], the BNP and SuNP in the west, and to a few large blocks of forest in the east. High human population density and extensive deforestation for agricultural practices [79] led to inadequate cover and low prey availability and tigers became extirpated in the eastern Terai region by the 1970s [80].With the eradication of malaria and the government policy of extending settlements along the border with India, large swaths of forest landscape were disturbed [81]. Taken together, these events resulted in the loss and fragmentation of the forest biomes in the TAL, likely causing the decline in tiger population size and genetic diversity, and the subsequent sub-structuring of the tiger population in Nepal, perhaps through population bottlenecks [36].

High rates of anthropogenic transformation of landscapes [36] play a major role in the extinction of wild mammal populations [82]. Intense forest fragmentation has imposed similar effects upon jaguars in Brazil [83] and Amur leopards in the Russian Far East [84], causing significant loss of genetic variation. In contrast, tigers [27, 85] and leopards [86] in India have high levels of genetic variation, and no genetic bottlenecks have been detected despite habitat fragmentation.

### Sampling strategy

Our sampling strategy was to collect as many as possible from the potential habitat (protected areas, surrounding buffer zones forests, and corridors) along TAL. We screened the duplication of samples at two stages: at field, we only collected the fresh scat samples at each encounter and avoided collection of the same scats samples by putting in the red marker on the scat (dried) itself and/or placed a twig into soil at the site of fresh samples. At the analysis phase, each of the duplicate samples (if scored) were taken as recapture.

### Genetic variation in TAL

Tigers in the TAL displayed moderate levels of genetic variation (H_e_ = 0.61) across the landscape and similar levels across sub-populations (H_e_ ≈ 0.57), perhaps due to moderate population size and/or gene flow resulting from potential connectivity among tiger-bearing protected areas across the TAL in Nepal (*n* = 5) and India (*n* = 10).Our estimates of genetic variation were lower in comparison to genetic diversity estimates for Bengal tigers overall (H_e_ = 0.72) [87] or the Indian Subcontinent (H_e_ = 0.70) [22] using microsatellite loci. However, the estimates were higher than in other sub-species of tigers (average H_e_ = 0.53, Sumatran, Indochinese, Malayan and Siberian) [87]. Landscape-wide genetic variation in tigers across the major tiger conservation landscapes in India range from as low as H_e_ = 0.58 in the southeastern Ghats [88], H_e_ = 0.67 in the northeast landscape [89], H_e_ = 0.76 in the Western Ghats [24], and as high as H_e_ = 0.81 in the central Indian landscape [27]. However, care should be taken in interpretation of heterozygosity because direct comparisons of diversity are not possible since these studies employed different numbers and combinations of microsatellite loci.

The average inbreeding coefficient across the sub-populations in the TAL-Nepal suggests weak inbreeding that was statistically non-significant (*P* = 0.42). At the sub-population level, SuNP showed a weak sign of local inbreeding (F_IS_ = 0.16), which approached statistical significance (*P* = 0.06). This suggests the importance of connectivity in averting inbreeding depression in the small SuNP sub-population[90]. Spatially, SuNP is surrounded by human settlements, but retains dispersal potential through the northern and southern sections of the reserve. However, we did not detect any migrants into this population, but rather detected three migrants leaving this subpopulation. Additionally, the creation of the Pilibhit Tiger Reserve (India) to the south strengthens the possibility of future connectivity via tiger dispersal through the Lagga Bagga Forest in India (Dr A.J.T Johnsingh: personal communication). Currently, the net migration estimates suggest that more tigers are leaving SuNP than are immigrating (Fig 7).

### Genetic structure

Both population- and individual-based tests for assessing the level of genetic sub-division revealed moderate levels of differentiation across the landscape. This is in concordant with AMOVA and fixation index (F_st_) results where populations (sites) observed moderate differentiation showing three genetic clusters confirming that total population in Terai Arc may not be total panmixia. Our Bayesian clustering results support three distinct genetic clusters (sub-populations) within the TAL-Nepal, representing the three tiger bearing protected areas (CNP, SuNP, BNP) confirming our *a priori* hypothesis. Our field data suggested demographic contiguity in and around these protected areas [30]. However, with the absence of tiger samples from the Indian side of the TAL, there is a lack of clarity in explaining the overall genetic structure of tigers across the entire landscape; tigers could travel through the contiguous protected areas of India into Nepal. Joint camera trap surveys conducted across the transboundary protected areas revealed 10 common tigers dispersing between Nepal and India [91]. We did detect a strong isolation-by-distance effect for individual tigers across the TAL. Spatial auto-correlation analysis detected genetic spatial structuring at geographic scale of 75–100 km across all samples indicating high genetic association among individuals even at such broad distances, and gene flow for the tigers might be possible through the landscape. The Bayesian clustering analysis in STRUCTURE and migration analysis in BayesAss+, both assessing contemporary gene flow levels [92, 93], showed higher connectivity between SuNP and BNP than between BNP and CNP. In contrast F_ST_/D_EST_ showed higher connectivity between CNP and BNP, which are farther apart, than between SuNP and BNP, which are closer together.

### Gene flow and detection of migrants

The results of the assignment tests offered evidence of contemporary gene flow between tiger sub-populations suggesting that tigers can, and do, disperse across the TAL-Nepal. We detected 7 migrants (5 males, 2 females) moving between the sub-populations. Tigers are known to disperse long distances (~200 km) based on long-term camera-trap data [94]. Furthermore, dispersal has been recorded as far as 600 km in the central Indian landscapes [25]. Consequently, the detection of tiger dispersal (first-generation migrants) between protected areas, which range from 314 km (CNP and BNP) to 136 km (BNP and SuNP) apart, is expected if the landscape provides stepping stone habitats to allow tiger movement throughout the landscape.

Results of the BayesAss+ analysis showed high estimates of recent migration of tigers among the sub-populations. High net immigration rates were detected in BNP. In the medium and large sub-populations (BNP and CNP), we found evidence of one to two male tigers dispersing between the populations. There was also evidence of one male and two females moving from SuNP to BNP. If these tigers breed, this will likely avert the detrimental effects of inbreeding depression, thus flattening the slopes of extinctions curves accordingly [90]. Results from the STRUCTURE analyses (Table 5) detected 3–7 individuals with mixed ancestry suggesting that migrants have successfully reproduced in earlier generations.

The SuNP population, while small, holds the highest density of tigers, likely due to high prey density [19]. This could help explain why SuNP is the source of many emigrants. However, it could be argued that, due to the small size of the reserve and the high tiger density, there might not be space for dispersing male tigers to establish territories within the reserve. Hence, it may not be possible to receive migrants from surrounding areas. Lower levels of genetic variation, and the slight but weak evidence of inbreeding relative to other protected areas in the landscape, suggests the need to increase migration of tigers into the SuNP population to avert inbreeding depression and increase genetic variation of the subpopulation. For example, leopards in the Russian Far East suffered significant loss in genetic variation due to lack of connectivity to a source population and continue to suffer loss of genetic variation [84]. The designation of 727 km^2^ of the Pilibhit Forest Division as a tiger reserve by the Indian government is an important step towards increasing connectivity between the western TAL in India and SuNP in Nepal (Dr. Dipankar Gosh WWF India: personal communication).

### Population bottlenecks

While both analysis techniques revealed population bottlenecks in the TAL-Nepal, there was a disagreement in the resulting number of detected population bottlenecks. The M-ratio test revealed a population bottleneck in all populations, in contrast to the heterozygous excess test showing a bottleneck only in the CNP population. Both tests have been found to be effective at detecting bottlenecks, but each works under different assumptions. Peery et al. [95] suggested that despite correctly assuming the mutation models (IAM, SSM or TPM), statistical power to detect a bottleneck with the two methods might depend upon the pre-bottleneck genetic variation. Heterozygosity may be less powerful than the M-ratio test when pre-bottleneck genetic diversity is high [95]. Alternatively, the heterozygosity excess test may work best when the pre-bottleneck population is smaller or when the bottleneck is milder and more recent[96]. Either way, there is evidence that at least one bottleneck occurred in the TAL-Nepal.

## Conclusions

We provide evidence of three genetically admixed sub-populations across the TAL-Nepal based on spatial (TESS) and non-spatial (STRUCTURE) Bayesian clustering techniques, suggesting that tigers have been able to move between the populations and breed, at least in the recent past. Contemporary gene flow measures of tigers were estimated in the TAL based on both likelihood and Bayesian approaches. Improved connectivity between the protected areas, facilitated by male and female tiger dispersal, appears to be able to avert the negative consequences of inbreeding depression following bottleneck events. Thus, there is a need to maintain connectivity throughout the TAL-Nepal landscape and beyond. We did not find migrants from CNP into SuNP or vice versa in any of the migrant detection tests. However, the dispersal among SuNP and CNP is more likely to be a stepping-stone process [97].

In recent times, connectivity in the Nepali landscape has been improved by the protection of large forest blocks after the nationalization of forests in the 1960s [77], improved governance, and management of forest resources [98]. The launch of the community forest program in the “Terai forest” [99] in the early 1980s has been successful in building more forest habitat at forest edges as well as buffers for the large forest blocks with the goal to increase tiger dispersal across the landscape. The Government of Nepal (GoN) has also taken positive steps in restoring connectivity across the TAL, including the adoption of a successful, community-based forest management approach [29]. The GoN’s declaration of all the identified forest corridor as a “protected forest” status was also a milestone [100]. Our results indicate that the landscape is currently functional with respect to the dispersal of tigers among the protected areas, but there is evidence of genetic structure, indicating that sub-populations exist and gene flow is limited between some protected areas. In the face of human population growth, economic development post-insurgency, political unrest, and developmental road projects, genetic connectivity seems likely to erode [101]. Consequently, gene flow of tigers across the landscape will be impeded, thus lowering their persistence in the long run [90]. Therefore, it is essential to secure tiger core areas and functional forest corridors between them to maintain and enhance gene flow across the landscape, thereby ensuring that tiger populations exist for generations to come. Securing tiger existence across the TAL-Nepal will require concerted stakeholder cooperation, careful planning, and the prevention of detrimental development activities.

## Supporting information

S1 Table Primers information for tiger specific species and sex identification. bp: base pair; F: forward; R: reverse.
(DOC)

S2 Table Thermo-cycling conditions for tiger species and sex identification PCR. PCR: polymerase chain reaction; min: minute; sec: second; “x” indicates times; F: forward; R: reverse.
(DOC)

S3 Table Genetic variability of 17 candidate microsatellite loci screened and “*” indicates loci used in this study. N_A_, number of alleles; H_o_, observed heterozygosity.
(DOC)

S4 Table Summary results from analysis of molecular variance (AMOVA) for tigers detected across three populations across the Terai Arc Landscape implemented in program ARLEQUIN 3.5 [53]. df = degree of freedom, *P* value (α = 0.05).
(DOC)

S5 Table Summary of pair-wise migration rate (immigration and emigration) between three populations estimated in Program BayesAss+[70]. Net migration rates (immigration-emigration) were estimated as 0.02 for CNP, -0.08 for SWR, and +0.10 for BNP; “+” indicate migrant receiving from other population; “-” indicate contributing migrant to other population.
(DOC)

S6 Table Results from program Bottleneck showing the expected and actual numbers of loci with heterozygosity excess under the respective mutation models, and significance of heterozygosity excess. IAM: Infinite Allele Model; TPM: Two-Phase Mutation Model; SMM: Stepwise-Mutation Model. Assuming any mutation model, a Wilcoxon test results with *P*<0.05 signifies significant heterozygous excess, suggesting that a bottleneck event occurred in CNP only.
(DOC)

S1 Fig Century-wide land-use change detected in the Terai Arc using Anthrome 2.0 datasets[36]. (TIF)

S2 Fig Magnitude of *∆k±* SD (rate of change in the log probability of *k; *SD: standard deviation) and Ln P(*k*) ± SD (posterior probability of the data; SD: standard deviation) as a function of *k*(number of sub-populations) detected three and four genetic clusters in the sampled population following [60]. (TIF)

S3 Fig STRUCTURE bar plot at k = 2 (top) and k = 4 (bottom) visualizing individual-based genetic differentiation in tigers across the Terai landscape, Nepal. (TIF)

S4 Fig Optimal number of genetic clusters (k_max_) based on DIC (Deviance Information Criteria) for admixture models (CAR and BYM). Both models selected three genetic clusters across the landscape. Error bars represent standard deviations.
(TIF)

S5 Fig Spatial autocorrelogram for *Panthera tigris tigris* in the Terai Arc Landscape, Nepal. The spatial correlogram for tigers (*n* = 78) shows the genetic correlation coefficient (*r*) as a function of geographic distance across defined spatial distance classes. Dashed red lines represent upper (U) and lower (L) bounds of the null hypothesis based on 9,999 random permutations. Error bars represent 95% confidence intervals about *r* based on 999 bootstraps.
(TIF)
