# Supplementary material for: Assessment of genetic diversity, population structure, and gene flow of tigers (Panthera tigris tigris) across Nepal's Terai Arc Landscape
Source: PLoS One. 2018 Mar 21;13(3):e0193495. doi: 10.1371/journal.pone.0193495 (PMC5862458; doi:10.1371/journal.pone.0193495)
Supplement: S1 Table — bp: base pair; F: forward; R: reverse. (DOC) [file pone.0193495.s001.doc]

**S1Table:** Primers information for tiger specific species and sex identification. bp: base pair; F: forward; R: reverse.

| Primer Name | Sequence (5’-3’) | Melting Temp | Product size | Reference |
| --- | --- | --- | --- | --- |
| TIF | ATAAAAAATCAGGAATGGTG | 55^0^C | 162 bp | Bhagavatula& Singh (2006) |
| TIR | TGGCGGGGATGTAGTTATCA | 65^0^C |
| AMEL-F | CGAGGTAATTTTTCTGTTTACT | 55^0^C | 194 bp& 214 bp (male); 214 bp (female) | Pilgrim et al. 2005 |
| AMEL-R | GAAACTGAGTCAGAGAGGC | 57.3^0^C |
| CYTB-SCT-F | AAACTGCAGCCCCTCAGAA  TGATATTTGTCCTCA | 72.9^0^C | 150 bp | Janecka et al. 2008 |
