# Supplementary material for: Assessment of genetic diversity, population structure, and gene flow of tigers (Panthera tigris tigris) across Nepal's Terai Arc Landscape
Source: PLoS One. 2018 Mar 21;13(3):e0193495. doi: 10.1371/journal.pone.0193495 (PMC5862458; doi:10.1371/journal.pone.0193495)
Supplement: S2 Table — PCR: polymerase chain reaction; min: minute; sec: second; “x” indicates times; F: forward; R: reverse. (DOC) [file pone.0193495.s002.doc]

**S2 Table** Thermo-cycling conditions for tiger species and sex identification PCR

| **Steps** | **Species PCR**  **(TI-F/R)** | | | **Sex PCR**  **(AMEL –F/R)** | | | **Cytochrome B PCR**  **(CYTB-SCT-F/R)** | | |
| --- | --- | --- | --- | --- | --- | --- | --- | --- | --- |
|  | **Temperature** | **Time** | **Cycling** | **Temperature** | **Time** | **Cycling** | **Temperature** | **Time** | **Cycling** |
| Initial denaturation | 95°C | 15 mins | 1X | 95°C | 15 mins | 1X | 95°C | 15 mins | 1X |
| Denaturation | 94°C | 30 sec | 35X | 94°C | 30 sec | 45X | 94°C | 15 sec | 45X |
| Annealing | 59°C | 90 sec |  | 53°C | 1 min |  | 55°C | 30 sec |  |
| Extension | 72°C | 90 sec |  | 72°C | 1 min |  | 72°C | 1 min |  |
| Final Extension | 72°C | 10 mins | 1X | 72°C | 10 mins | 1X | 72°C | 5 mins | 1X |
| Hold | 4°C | 30 mins | 1X | 4°C | hold | 1X | 4°C | hold | 1X |

(PCR: polymerase chain reaction; min: minutes; sec: second “x” indicates times; F: forward; R: reverse.)
