# Supplementary material for: Assessment of genetic diversity, population structure, and gene flow of tigers (Panthera tigris tigris) across Nepal's Terai Arc Landscape
Source: PLoS One. 2018 Mar 21;13(3):e0193495. doi: 10.1371/journal.pone.0193495 (PMC5862458; doi:10.1371/journal.pone.0193495)
Supplement: S3 Table — NA, number of alleles; Ho, observed heterozygosity. (DOC) [file pone.0193495.s003.doc]

**S3 Table** Genetic variability of 17 candidate microsatellite loci screened and “*” indicates loci used in this study. N_A_, number of alleles; H_O_, observed heterozygosity.

| **Locus** | **Allele Size Range (bp)** | **Repeat type** | **Ho** | **N_A_** | **Source** |
| --- | --- | --- | --- | --- | --- |
| Microsatellite Primer | | | | | |
| FCA205* | 102-116 | DI | 0.78 | 5 | Menotti-Raymond et al.1999 |
| FCA232* | 100-108 | DI | 0.44 | 3 | Menotti-Raymond et al.1999 |
| FCA272 | 115-127 | Di | 0.667 | 6 | Menotti-Raymond et al.1999 |
| FCA304* | 122-140 | Di | 0.622 | 5 | Menotti-Raymond et al.1999 |
| FCA391* | 122-154 | Tetra | 0.64 | 6 | Mondol et al.2009a |
| FCA441* | 78-118 | Tetra | 0.57 | 4 | Mondol et al.2009a |
| FCA453 | 69-89 | Tetra | 0.43 | 5 | Mondol et al.2009a |
| FCA742 | 160-184 | Tetra | 0.55 | 6 | Menotti-Raymond et al.1999 |
| F41 | 100-172 | Tetra | 0.429 | 4 | Mondol et al.2009a |
| F42 | 152-180 | Tetra | 0.43 | 5 | Mondol et al.2009a |
| F53* | (165 - 181) | Tetra | 0.778 | 5 | Menotti-Raymond et al.1999 |
| F85* | 155-167 | Tetra | 0.6 | 4 | Menotti-Raymond et al.1999 |
| PttD5* | 200-224 | Tetra | 0.61 | 4 | Mondol et al.2009a |
| PttA2 | 188 - 198 | DI | 0.51 | 4 | Mondol et al.2009a |
| Pun82 | 105-123 | DI | 0.483 | 5 | Janecka et al. 2008 |
| FCA043* | 111-117 | DI | 0.52 | 6 | Menotti-Raymond et al.1999 |
| FCA008 | 128-140 | DI | 0.55 | 4 | Menotti-Raymond et al.1999 |
