# Supplementary material for: Assessment of genetic diversity, population structure, and gene flow of tigers (Panthera tigris tigris) across Nepal's Terai Arc Landscape
Source: PLoS One. 2018 Mar 21;13(3):e0193495. doi: 10.1371/journal.pone.0193495 (PMC5862458; doi:10.1371/journal.pone.0193495)
Supplement: S4 Table — df = degree of freedom, P value (α = 0.05). (DOC) [file pone.0193495.s004.doc]

**S4 Table** Summary results from analysis of molecular variance (AMOVA) for tigers detected across three populations across the Terai Arc Landscape implemented in program ARLEQUIN 3.5 (Excoffier and Lischer 2010). df = degree of freedom, P value (α=0.05)

| **Source of Variation** | **df** | **Sum of Squares** | **Variance Component** | **Percentage of Variation** | ***P* Value** |
| --- | --- | --- | --- | --- | --- |
| Among Populations | 2 | 32.155 | 0.28998 | 13.72 | <0.00 |
| Within Populations | 153 | 279.031 | 1.82373 | 86.28 | <0.00 |
| Total | 155 | 311.186 | 2.11371 |  |  |
