# Supplementary material for: Assessment of genetic diversity, population structure, and gene flow of tigers (Panthera tigris tigris) across Nepal's Terai Arc Landscape
Source: PLoS One. 2018 Mar 21;13(3):e0193495. doi: 10.1371/journal.pone.0193495 (PMC5862458; doi:10.1371/journal.pone.0193495)
Supplement: S5 Table — Net migration rates (immigration-emigration) were estimated as 0.02 for CNP, -0.08 for SWR, and +0.10 for BNP; “+” indicate migrant receiving from other population; “-” indicate contributing migrant to other population. (DOC) [file pone.0193495.s005.doc]

**S5 Table Summary of pair-wise migration rate (immigration and emigration) between three populations estimated in Program BayesAss+.**Net migration rates (immigration-emigration) were estimated as 0.02 for CNP, -0.08 for SWR, and +0.10 for BNP

|  | **Population** | **Migrating from** | | |
| --- | --- | --- | --- | --- |
|  |  | CNP | BNP | SWR |
| **Migrating**  **into** | CNP | 0.98 | 0.01 | 0.01 |
|  | BNP | 0.04 | 0.83 | 0.13 |
|  | SWR | 0.02 | 0.02 | 0.96 |
