# Supplementary material for: Assessment of genetic diversity, population structure, and gene flow of tigers (Panthera tigris tigris) across Nepal's Terai Arc Landscape
Source: PLoS One. 2018 Mar 21;13(3):e0193495. doi: 10.1371/journal.pone.0193495 (PMC5862458; doi:10.1371/journal.pone.0193495)
Supplement: S6 Table — IAM: Infinite Allele Model; TPM: Two-Phase Mutation Model; SMM: Stepwise-Mutation Model. Assuming any mutation model, a Wilcoxon test results with P<0.05 signifies significant heterozygous excess, suggesting that a bottleneck event occurred in CNP only. (DOC) [file pone.0193495.s006.doc]

**S6 Table** Results from program Bottleneck showing the expected and actual numbers of loci with heterozygosity excess under the respective mutation models, and significance of heterozygosity excess: IAM: Infinite Allele Model; TPM: Two-Phase Mutation Model; SMM: Stepwise-Mutation Model. Assuming any mutation model, a Wilcoxon test results with P<0.05 signifies significant heterozygous excess, suggesting that a bottleneck event occurred in CNP only

| **Population** | **Heterozygote excess** | **IAM** | **TPM** | **SMM** |
| --- | --- | --- | --- | --- |
| CNP | Expected | 4.53 | 4.71 | 4.77 |
|  | Actual | 6 | 5 | 3 |
|  | *p* value | *0.02* | 0.62 | 0.80 |
| BNP | Expected | 4.59 | 4.72 | 4.74 |
|  | Actual | 5 | 3 | 3 |
|  | *p* value | 0.23 | 0.87 | 0.87 |
| SWR | Expected | 4.33 | 4.67 | 4.65 |
|  | Actual | 7 | 6 | 6 |
|  | *p* value | 0.09 | 0.23 | 0.23 |
