# Supplementary figures and images for: Assessment of genetic diversity, population structure, and gene flow of tigers (Panthera tigris tigris) across Nepal's Terai Arc Landscape
Source: PLoS One. 2018 Mar 21;13(3):e0193495. doi: 10.1371/journal.pone.0193495 (PMC5862458; doi:10.1371/journal.pone.0193495)

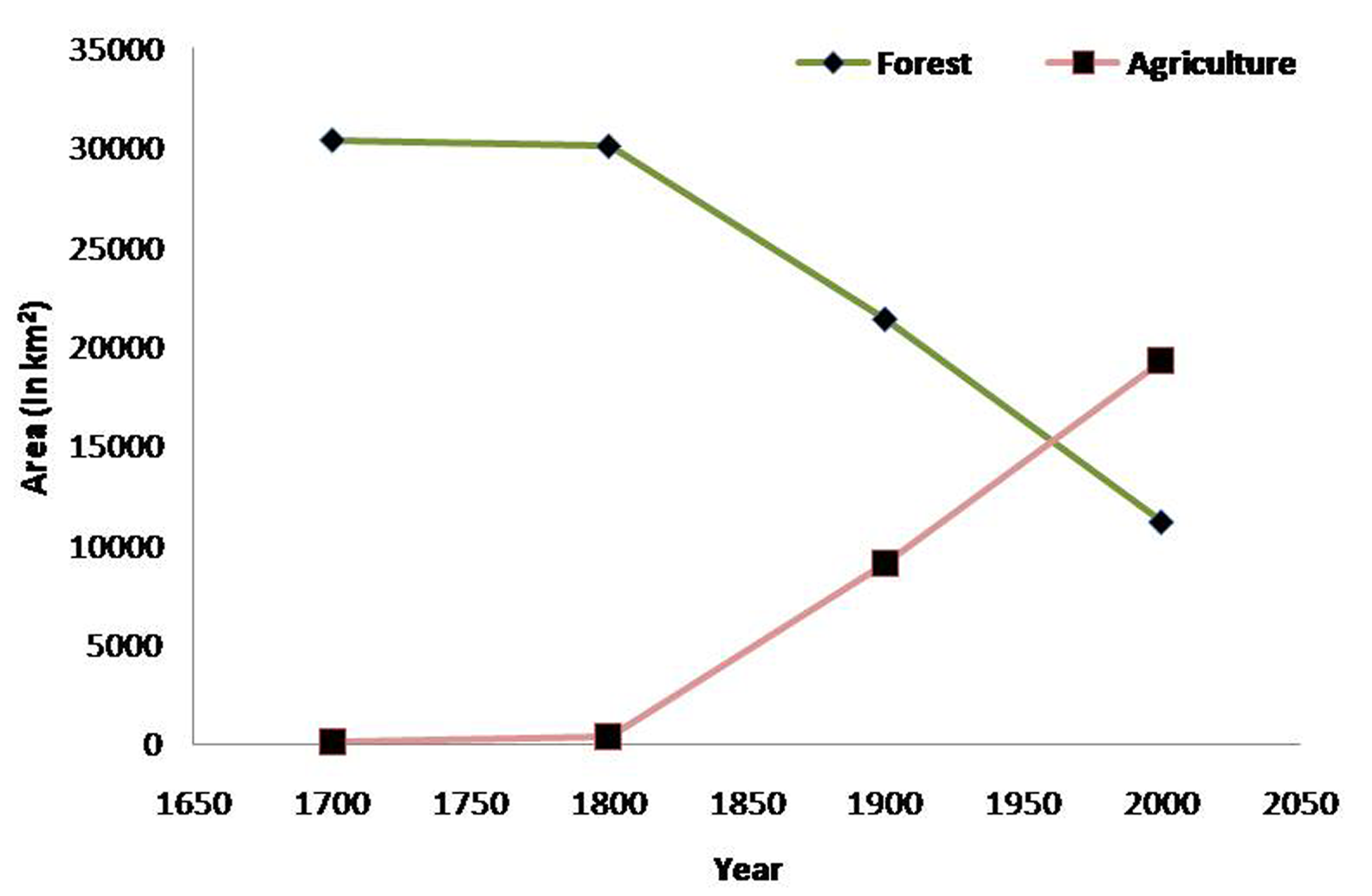

Supplement: S1 Fig — (TIF) [file pone.0193495.s007.tif]

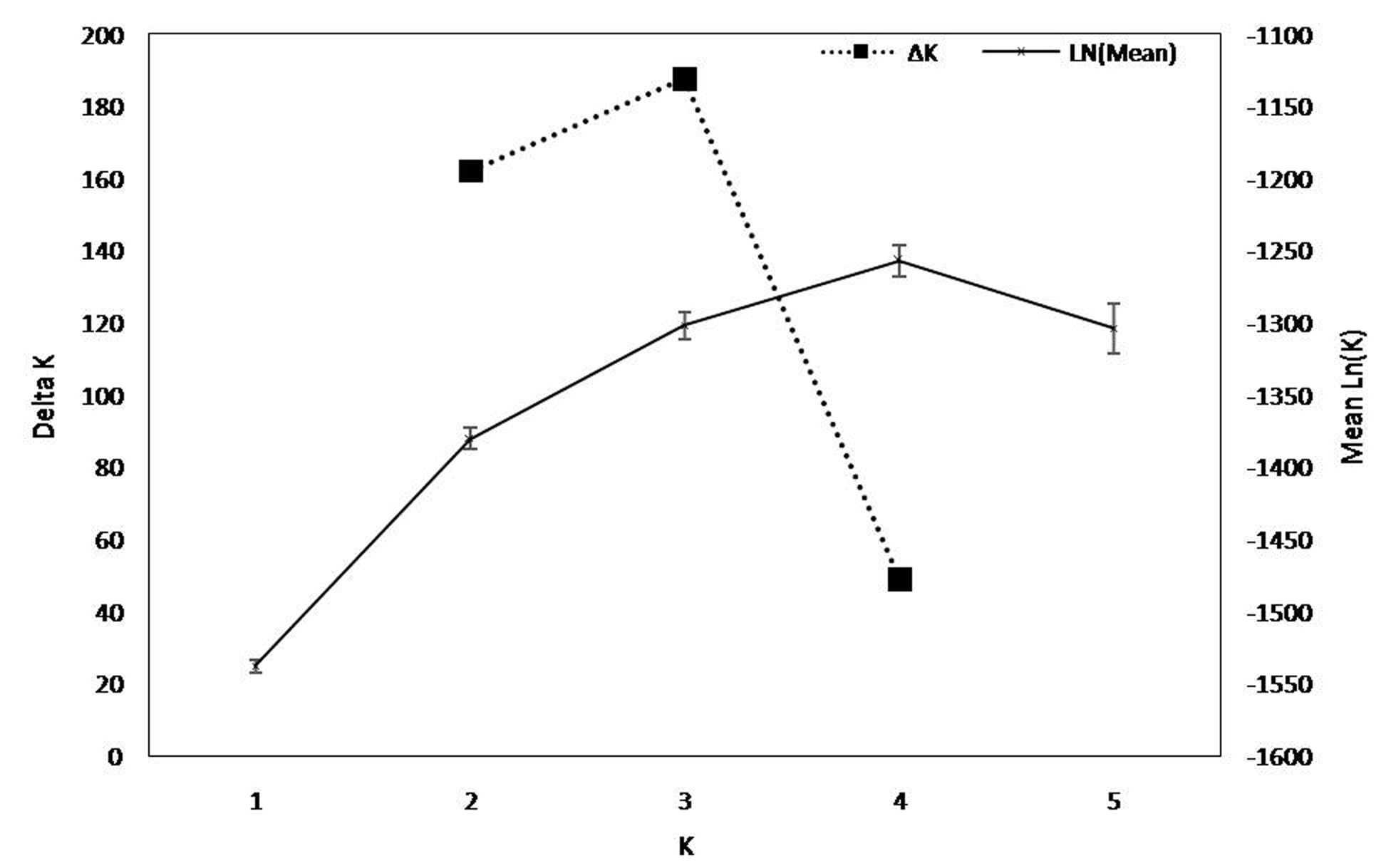

Supplement: S2 Fig — (TIF) [file pone.0193495.s008.tif]

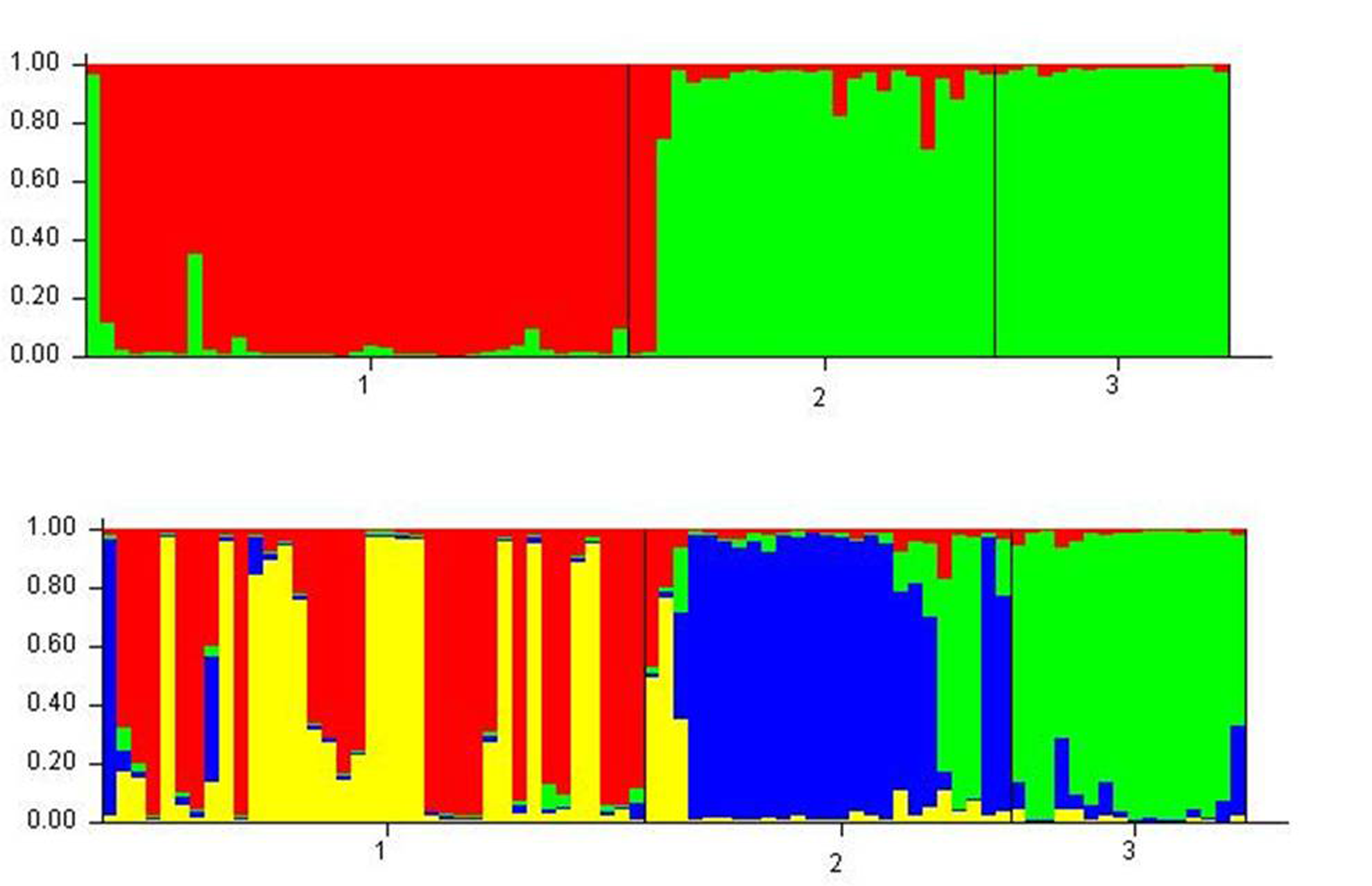

Supplement: S3 Fig — (TIF) [file pone.0193495.s009.tif]

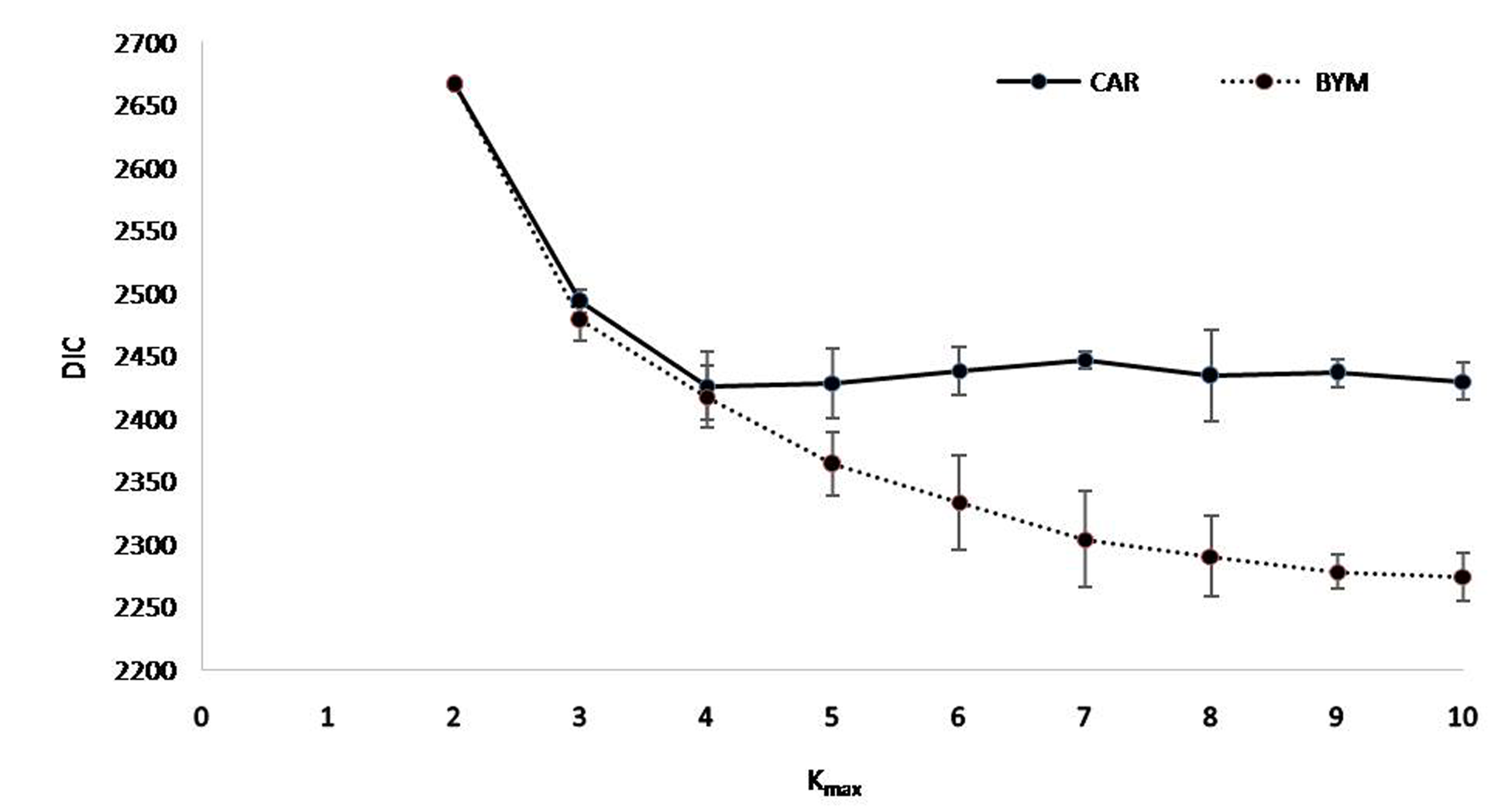

Supplement: S4 Fig — Both models selected three genetic clusters across the landscape. Error bars represent standard deviations. (TIF) [file pone.0193495.s010.tif]

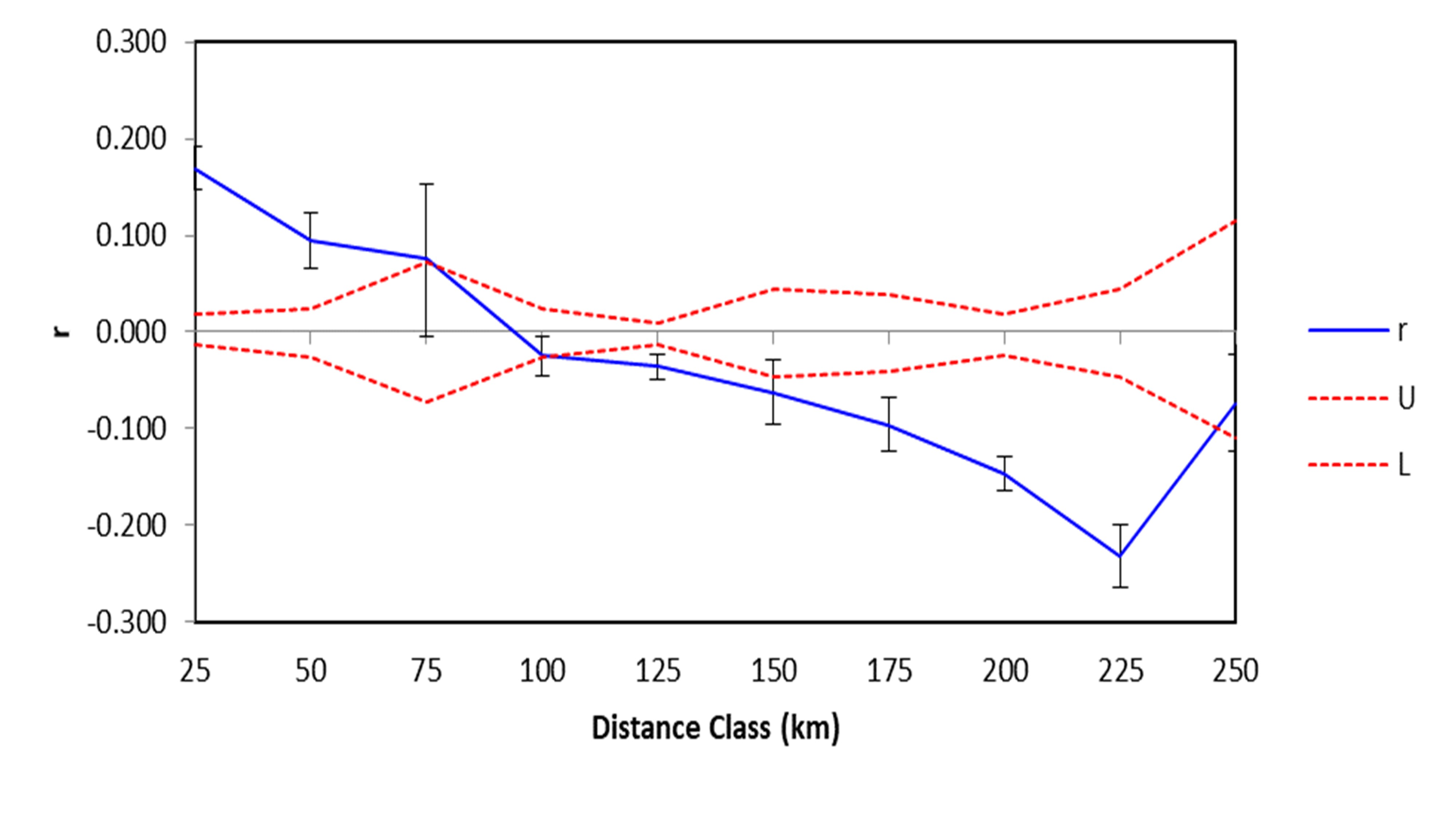

Supplement: S5 Fig — The spatial correlogram for tigers (n = 78) shows the genetic correlation coefficient (r) as a function of geographic distance across defined spatial distance classes. Dashed red lines represent upper (U) and lower (L) bounds of the null hypothesis based on 9,999 random permutations. Error bars represent 95% confidence intervals about r based on 999 bootstraps. (TIF) [file pone.0193495.s011.tif]
